# Similar quality of life after balloon pulmonary angioplasty or pulmonary endarterectomy for CTEPH

**DOI:** 10.1016/j.jhlto.2025.100223

**Published:** 2025-01-31

**Authors:** P. Smeele, N.J. Braams, L. Vermunt, A. Kianzad, T.C. Rodenburg, E. Stilling, S. Andersen, M.J. Andersen, S. Mellemkjær, E.J. Nossent, Jurjan Aman, M.A.M. Beijk, R.J. Lely, J.A. Winkelman, F. Oosterveer, T. Marcus, A. Vonk Noordegraaf, L.J. Meijboom, C.E. Teunissen, A. Andersen, H.J. Bogaard

**Affiliations:** a Department of Pulmonary Medicine, Amsterdam University Medical Centre, Amsterdam, the Netherlands; b Neurochemistry Laboratory, Department of Laboratory Medicine, Amsterdam Neuroscience, Amsterdam University Medical Centre, Vrije Universiteit, Amsterdam, the Netherlands; c Department of Cardiology, Aarhus University Hospital, Aarhus, Denmark; d Department of Cardiology, Amsterdam University Medical Centre, Amsterdam, the Netherlands; e Department of Radiology, Amsterdam University Medical Centre, Amsterdam, the Netherlands; f Department of Cardiothoracic Surgery, Amsterdam University Medical Centre, Amsterdam, the Netherlands; g Cardiovascular Sciences Research Institute, Amsterdam, the Netherlands

**Keywords:** chronic thromboembolic pulmonary hypertension (CTEPH), balloon pulmonary angioplasty (BPA), pulmonary endarterectomy (PEA), living with pulmonary hypertension questionnaire (LPHQ), quality of life (QoL)

## Abstract

**Background:**

Chronic thromboembolic pulmonary hypertension (CTEPH) is most commonly treated with pulmonary endarterectomy (PEA) or with a combination of pulmonary vasodilators and balloon pulmonary angioplasty (BPA). Quality of life (QoL) after treatment is one of many factors used to determine which procedure is most suitable for each patient. Studies evaluating QoL after these interventions have not compared QoL between both procedures in a randomized cohort. In this prospective registry study, we explored QoL as an outcome in patients treated with PEA or BPA treatment and which factors correlated to QoL.

**Methods:**

CTEPH patients treated in Amsterdam University Medical Center and Aarhus University Hospital, the living with pulmonary hypertension questionnaire (LPHQ) was recorded to assess QoL before treatment and 6 months after treatment. Multiple pulmonary and hemodynamic parameters were recorded as part of standard clinical care.

**Results:**

At baseline 99 LPHQ questionnaires were answered and 67 at 6 months. Baseline parameters did not differ significantly between patients undergoing PEA vs BPA. QoL was similar at baseline in both treatment groups, and both groups experienced a similar improvement in QoL after treatment. The New York Heart Association (NYHA) score and Borg score after the six-minute walking distance (6MWD) were most strongly correlated to QoL at all time points. Baseline carbon monoxide diffusion capacity (DLCO) was inversely correlated to QoL after treatment. Presence of residual PH was not associated with significantly decreased QoL.

**Conclusions:**

We did not find evidence that QoL differed in 2 separate groups of patients who underwent either BPA or PEA. Multiple parameters pertaining to dyspnea and basic functionality were moderately correlated to QoL, this was not the case for hemodynamic parameters. These results indicate that when determining invasive treatment in CTEPH, QoL can be expected to improve with both treatments.

**Take Home Message**

Quality of Life (QoL) in patients with chronic thromboembolic pulmonary hypertension (CTEPH) improves to a similar degree after pulmonary endarterectomy and balloon pulmonary angioplasty. QoL correlated with parameters for dyspnea and basic functionality at all time points and improvement of certain hemodynamic parameters was associated to improvement of QoL. A low carbon monoxide transfer factor (DLCO) at baseline was predictive of poor QoL after treatment. Interestingly, QoL was similar in patients with or without residual PH.

Chronic thromboembolic pulmonary hypertension (CTEPH) is characterized by exertional dyspnea and reduced fitness. It is a form of pulmonary hypertension (PH) that results from persistent obstruction of the pulmonary vascular bed due to residual organized thrombi, often after pulmonary embolism. Patients experience reduced functionality and reduced participation in daily activities. Of all the causes of pulmonary hypertension, CTEPH is unique in that curation is possible.^1^

Pulmonary endarterectomy (PEA) is currently considered the gold standard concerning treatment modalities in eligible patients, showing high survival rates with hemodynamic improvement and improved quality of life (QoL) in the majority of patients.^2^, ^3^, ^4^, ^5^, ^6^, ^7^, ^8^, ^9^ Balloon pulmonary angioplasty (BPA), often in combination with pulmonary vasodilator therapy, appears to improve outcomes to a similar degree as PEA, with most published reports focusing on survival, hemodynamic recovery and QoL.^10^, ^11^, ^12^, ^13^, ^14^ Comparisons of QoL outcomes between these 2 treatment modalities have been limited by small sample sizes, the differing eligibility for surgery among patients and the use of non-specific QoL questionnaires that have not been validated in PH.^15^ Furthermore, the exploration of potential predictors of improved QoL after therapy are limited.

Patients with CTEPH experience a substantially reduced QoL compared to the general population. Anxiety, depression, and social isolation due to a lack of understanding by family and friends have been reported.^16^, ^17^, ^18^ To address this, multiple Patient reported outcome measures (PROMs) have been used to study QoL in patients with CTEPH. These range from generic QoL assessments such as the Rand 36-item Short Form (SF-36) and EuroQol-5D (EQ-5D) to PROMs validated in PH, particularly in pulmonary arterial hypertension (PAH) and CTEPH such as the CAMPHOR and the living with pulmonary hypertension questionnaire (LPHQ).^6^, ^19^, ^20^

Due to the fundamental differences between these therapies, both in risk and availability, and the increasing overlap in the patient populations that can avail of both treatments, it remains important to understand if and how PH-specific QoL outcomes differ after PEA and BPA. This will help to guide treatment decisions for patients whom are eligible for both surgery and radiologic intervention. In this study, we assessed QoL in PEA and BPA patients treated at Amsterdam University Medical Center (UMC) and Aarhus University Hospital both before and at 6 months after treatment, and evaluated the predictors of improved QoL.

## Methods

### Study design and patient selection

This substudy is part of the dual center Amsterdam/Aarhus, CTEPH/CTED Cohort study (2A3C). In this study, patients were selected from a prospective observational cohort study of adult patients diagnosed with CTEPH who underwent PEA or BPA between 2017 and 2024 in the tertiary referral centers for CTEPH in Aarhus University Hospital, Denmark and Amsterdam UMC center of expertise for pulmonary hypertension, The Netherlands. In both centers, diagnosis of CTEPH was established according to current ESC/ERS (European society of Cardiology, European Respirattory society) guidelines during multidisciplinary meetings (MDT) with pulmonologists, (interventional) cardiologists, (interventional) radiologists, internal vascular medicine specialists, and critical care specialists present.^1^, ^21^ First choice of treatment strategy was made by these dedicated CTEPH-teams during MDT. Residual PH at 6 months after treatment was defined as a mean pulmonary artery pressure (mPAP) > 20 mm Hg and pulmonary vascular resistance (PVR) ≥ 2 WU according to the revised hemodynamic definition of the 2022 ESC/ERS Guidelines for the diagnosis and treatment of pulmonary hypertension.^1^ In both centers, patients were approached after screening and informed about the study. After a minimum of 24 hours of consideration, patients could consent to participate in the study. This study was approved by the Medical Ethics Review Committee of the Amsterdam UMC and Aarhus University Hospital (METC (medical ethics committee) reference – 2012.288) and performed in accordance with the declaration of Helsinki and according to Danish and Dutch regulations.

### Clinical parameters

Patients were followed according to a local standardized guideline-based protocol. They underwent follow-up measurements consisting of clinical and laboratory analysis, pulmonary function testing (PFT), six-minute walking distance (6MWD), right heart catheterization (RHC) and cardiopulmonary exercise test (CPET) before treatment and 6 months after treatment.

### Living with Pulmonary Hypertension Questionnaire

The validated “Living with Pulmonary Hypertension” is an adaptation of the Minnesota Living with Heart Failure survey.^19^ Both were initially validated in patients with pulmonary arterial hypertension and have been used in multiple large medical studies of patients with CTEPH.^22^, ^23^, ^24^ The questionnaire is composed of a total of 105 points which focus on physical, mental, and daily functioning aspects. Higher scores indicate a worse QoL. Patients received a translated version in their native language and filled this in manually while waiting between tests for routine clinical care. The questionnaire was answered in a quiet, private space. LPHQ data was collected pre-operatively and 6 months after therapy within a margin of +/- 1 month. The change in QoL was measured by subtracting LPHQ scores at 6 months from the baseline LPHQ score. A score of 0 points indicated that CTEPH did not hinder a patient in their daily life in the past 7 days.

### Statistical analysis

First, demographic characteristics were summarized and Student’s t-tests were used to identify baseline differences between both treatment groups (for the New York Heart Association [NYHA] score, Chi-square was used). Using histograms and Q-Q plots, variables were inspected and where needed log transformed for normality of distribution. The log transformation was applied to N-terminal pro b-type natriuretic peptide (NT-proBNP), mean right atrial pressure (mRAP) and PVR at both time points. Student’s *t*-tests were used to identify differences between QoL in both treatment groups at baseline, at 6 months, and in the delta between baseline and 6 months. After it was shown that there were no significant differences between therapy groups, all patients were grouped together for further analysis. Spearman’s correlation tests were used to assess the strength of correlations between LPHQ and all clinical variables at baseline and at 6 months. Furthermore, baseline variables were tested as predictors of LPHQ at 6 months. To assess which change in variables affected the change in LPHQ over time, linear mixed models were used where each variable was assessed as a covariate and a random intercept for participants was used. Results were ordered in ascending order of *p*-value and results were not corrected for multiple testing. Data is presented as *n* (%), mean with standard deviation (SD), or median with interquartile range (IQR) unless otherwise annotated. A *p*-value <0.05 was considered statistically significant. Statistical analysis was performed using R version 4.4.1.

## Results

**Figure 1:**
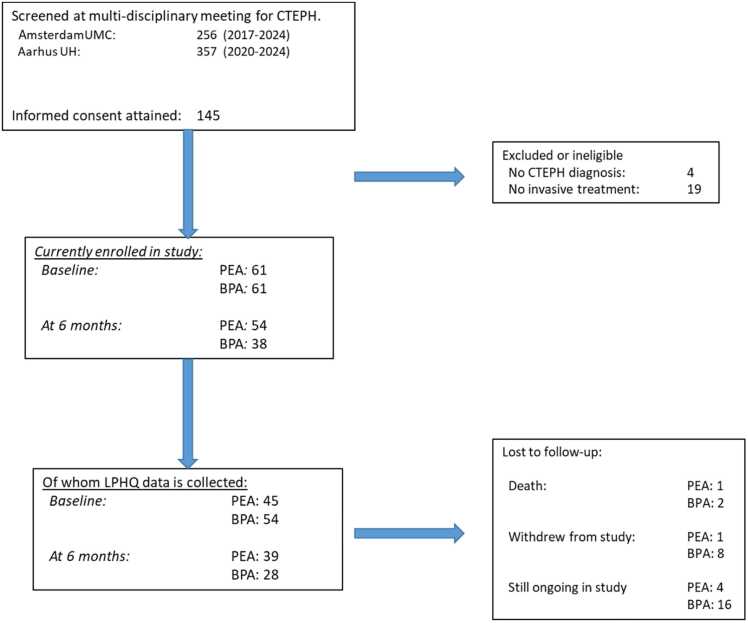
Overview of included patients in the 2A3C between both recruiting centers, Amsterdam UMC and Aarhus University Hospital. UMC, University Medical Center.

A total of 99 patients had LPHQ data recorded at baseline and 67 patients at 6 months (Figure 1). In total, 63 patients had corresponding data at both time points. The Amsterdam UMC cohort consisted of 60 patients of which 35 were treated with PEA and 25 treated with BPA. The Aarhus University hospital cohort consisted of 39 patients of whom 10 were treated with PEA and 29 with BPA. Apart from the BPA group being on average 4 years older, there were no significant differences in clinical, laboratory, lung function, hemodynamic and exercise tolerance parameters (Table 1). LPHQ scores did not differ at baseline between both research centers (data not shown).

**Table 1:** Demographic Characteristics of Cohort at Baseline

	BPA		PEA		*t*-value	*p*-value
*Demographics*						
*(N = BPA:54/PEA:45)*						
Age at time of consent	**67.1 (9.9)**		**62.7 (11.1)**		**2.08**	**0.04**
Gender						
BMI (kg/m^2^)	26.1 (4.5)		27.5 (4.5)		−1.5	0.14
Number of BPA sessions						
1−2	12					
3−4	19					
5−8	20					
Treatment still ongoing	3					
*Comorbidities*						
*(N = BPA:54/PEA:45)*						
COPD (chronic obstructive pulmonary disease)	4%		5%		0.54	0.103
Hypertension	22%		33%		−1.27	0.207
Ischemic heart disease	9%		4%		0.93	0.355
Kidney disease	4%		7%		−0.67	0.507
Other	45%		4%		0.54	0.588
None	38%		31%		0.74	0.464
*Medication*						
*(N = BPA:54/PEA:45)*						
Riociguat	**2%**	**48**	**13%**	**41**	**−2.12**	**0.039**
Sildenafil	4%	48	4%	41	−0.2	0.841
VKAs (vitamin K antagonists)	31%	48	47%	41	−1.61	0.111
NOAC (novel oral anticoagulants)	**69%**	**48**	**47%**	**41**	**2.29**	**0.024**
Loop diuretics	18%	48	16%	41	0.35	0.73
Aldosteron receptor antagonists	5%	48	2%	41	0.85	0.398
Others	5%	48	11%	41	−1	0.32
*Clinical, lab, LFT (lung function testing) and 6MWD*		N		N		
LPHQ (score out of 105)	36.6 (18.9)	54	40.6 (20.1)	45	−1.03	0.31
Δ LPHQ 6 months after therapy	21.3 (15)	27	25.7 (18.1)	35	−1.04	0.3
NYHA score	2.4 (0.6)	52	2.6 (0.6)	45	−1.45	0.15
DLCOc (mmol/(min*kPa))	6.3 (2.1)	30	6.4 (1.9)	35	−0.11	0.91
DLCOc (%pred)	70.1 (16.4)	46	72.5 (14)	39	−0.74	0.46
NTproBNP (ng/liter)	1,063.4 (1,517.9)	54	1,170.7 (1,610.6)	43	−0.33	0.74
6MWD (m)	438.2 (118.3)	47	444.2 (93.9)	30	−0.25	0.81
Borg score at end of exercise	5.5 (2.3)	46	5.4 (2.5)	28	0.17	0.86
*Right heart catheterization*						
*(N = 54/45)*						
mPAP (mm Hg)	39.3 (14.4)		41.7 (13.2)		−0.84	0.4
mRAP (mm Hg)	6.7 (4.1)		7.4 (3.8)		−0.87	0.39
Cardiac Index (l/min)	2.6 (0.7)		2.6 (0.7)		0.67	0.51
PAWP (mm Hg)	10.5 (3.5)		11 (2.7)		−0.77	0.45
PVR (dyn/s/cm^5^)	497.2 (314.4)		585.9 (411.7)		−1.19	0.24
SvO_2_ (%)	65.9 (10.2)		65.4 (7.9)		0.29	0.77
*Cardiopulmonary excercise testing*						
*(N = 47/41)*						
peakVO_2_ (%pred)	**71.4 (23.5)**		**61.5 (21.2)**		**2.09**	**0.04**
peakVO_2_ (ml/min)	1,349.5 (558.9)		1,280.1 (513.7)		0.61	0.54
peakVO_2_ (ml/kg/min)	16.4 (5.9)		14.7 (5.7)		1.34	0.18
Load max (watts)	105.1 (46.6)		99.1 (52.7)		0.56	0.58
Load_max (%pred)	**87.1 (39.9)**		**63 (32.1)**		**3.03**	**0.001**
Heart rate max (bpm)	130.4 (24.4)		133.5 (21.3)		−0.64	0.53
Heart rate max (%pred)	85.2 (14.8)		84.6 (12.5)		0.2	0.84
RERmax	3.4 (16)		1 (0.1)		1.02	0.31
O_2_pulse max (ml)	10.4 (3.4)		9.5 (3.4)		1.29	0.2
O_2_pulse max (%pred)	**83.8 (29.5)**		**70.5 (25.4)**		**2.27**	**0.03**
EqCO_2__AT	44 (11.8)		47.3 (10.6)		−1.33	0.19

6MWD, six minute walking distance; BMI, Body Mass Index; DLCOc, diffusing capacity of the lungs for carbon monoxide; EqCO_2__AT, carbon dioxide equivalent at anaerobic threshold; LPHQ, Living with Pulmonary Hypertension Questionnaire; mPAP, mean pulmonary artery pressure; mRAP, mean right atrial pressure; NTproBNP, N-terminal pro b-type natriuretic peptide; NYHA, New York Heart Association; PAWP, pulmonary artery wedge pressure; PVR, pulmonary vascular resistance; QoL, quality of life_._

Bold values represent values that significantly differed between treatment groups.

When examining the change in clinical parameters after treatment, the PEA group showed significantly more hemodynamic improvement, notably in the mPAP and PVR. A comprehensive overview of all baseline variables per treatment group is shown in Supplementary Table 1.

**Figure 2:**
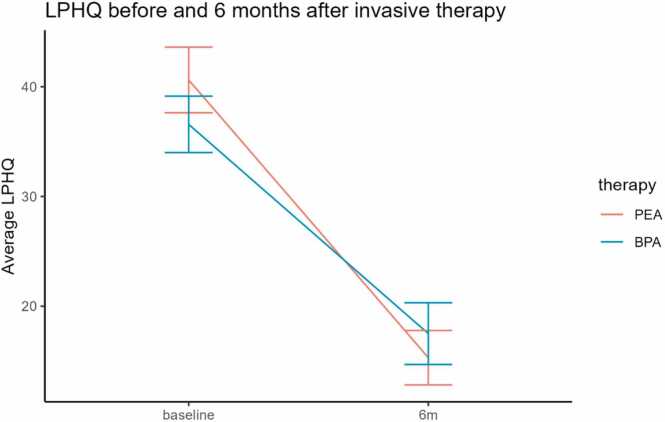
Line graph showing QoL improving in the first 6 months after treatment (lower LPHQ implies better QoL) Blue: Pulmonary endarterectomy group, Red: Balloon angioplasty group. See Table 3 for the number of patients per group and time point*.* LPHQ, living with pulmonary hypertension questionnaire; QoL, quality of life.

QoL did not differ between PEA patients and BPA patients at baseline or at 6 months after treatment. The PEA group had an LPHQ score of 40.6 (20.1) and recovered to an LPHQ score of 15.3 (15.5). The BPA group had a score of 36.5 (18.9) at baseline and recovered to 17.6 (15.1). The relative improvement (delta) of QoL did not differ significantly between groups, though a trend towards PEA patients was observed (Figure 2, Table 2).

**Table 2:** Overview of LPHQ Scores per Time Point per Therapy Group

Timepoint	BPA	PEA	*p*-value
Baseline			
N	54	45	
LPHQ score	36.5 (19)	40.6 (20)	0.31
6 months			
N	27	39	
LPHQ score	17.6 (15)	15.3 (15)	0.54
Δ LPHQ			
N	27	35	
Δ LPHQ score	21.3(15)	25.6 (18)	0.26

BPA, balloon pulmonary angioplasty; LPHQ, Living with Pulmonary Hypertension Questionnaire; N, number of patients per group; PEA, pulmonary endarterectomy.

Mean difference and *p-value calculated using student’s paired t-test. Delta LPHQ calculated using ANOVA. Analysis includes 99 at baseline, 66 patients at 6 months, and 62 patients who have both baseline and 6 months data.*

Since none of the outcomes differed between the PEA and BPA groups, subsequent analyses were made considering all patients as one group. At baseline, QoL correlated moderately with the NYHA score (rho: 0.380, *p*-value: 0.001), the 6MWD (rho: −0.349, *p*-value: 0.002) and the perceived dyspnea afterwards (Borg score, rho: 0.324, *p*-value: 0.005). These variables at baseline were also able to predict LPHQ at 6 months together with maximum oxygen saturation during the 6MWD and CPET test (rho 0.338 and 0.300 and *p*-value 0.027 and 0.03 respectively) and carbon monoxide transfer capacity (DLCO, rho −0.304 and *p*-value: 0.036) (Table 3). As it would be biologically implausible for a higher maximum oxygen saturation during 6MWD and CPET to correlate to a worse quality of life after treatment, and as the results were driven by outliers in the data, this result was not further explored. When the variables at 6 months were correlated to LPHQ at 6 months, NYHA and Borg score correlated again with QoL, together with C-reactive protein, cardiac output and peak VO_2_/kg (Supplementary Table 3).

**Table 3:** a and b Spearman Correlation Test Results Showing All Correlations with *p*-Value <0.05 of Baseline Predictors of LPHQ at Baseline (a), and All Correlations with *p*-Value <0.05 of Baseline Predictors to LPHQ at 6 Months (b)

(a)			
Predictors at baseline vs LPHQ at baseline	Correlation	*p*-value	N
NYHA	0.380	0.001	97
Six minute walking distance	−0.349	0.002	77
Borg score after 6MWD	0.324	0.005	74
peakVO_2_ (ml/kg/min)	−0.268	0.012	87
Heart rat% of max predicted	−0.260	0.014	88
Maximum ventilatory capacity, % predicted	−0.240	0.029	83
Maximum ventilatory capacity	−0.207	0.053	88

*(b)*			
*Predictors at baseline vs LPHQ at 6 months*	*Correlation*	*p-value*	*N*

Maximum O_2_% during six minute walking distance	0.338	0.027	43
Maximum O_2_% during cardiopulmonary exercise testing	0.300	0.030	52
DLCOc	−0.304	0.036	48
Borg score after 6MWD	0.319	0.042	41
Six minute walking distance	−0.304	0.042	44
NYHA	0.247	0.05	62
BMI	0.242	0.056	63

6MWD, six minute walking distance; BMI, Body Mass Index; DLCOc, diffusing capacity of the lungs for carbon monoxide; LPHQ, Living with Pulmonary Hypertension Questionnaire; NYHA, New York Heart Association; QoL, quality of life.

N represent the number of patients included in each analysis. Results not corrected for multiple testing. As LPHQ is an inverted score, a positive correlation indicates that an increase of the variable leads to a worse QoL.

The improvement in LPHQ over time, as tested using linear mixed models, was positively associated with improvements over time in the NYHA score, Borg score after 6MWD and peak VO_2_/kg (Table 4). A likelihood ratio test confirmed that using each variable as an interaction term did not improve the model accuracy.

**Table 4:** Showing Linear Mixed Models Illustrating the Variables Significantly Associated with Change in LPHQ Over Time

	Estimate	Std. error	*t*-value	*p*-value
NYHA	7.70	2.29	3.36	0.001
Borg score after 6MWD	2.34	0.80	2.91	0.005
peakVO_2_ (ml/kg/min)	−0.67	0.34	−1.96	0.053
SpO_2_max	0.74	0.43	1.71	0.091
SvO_2_	−0.33	0.19	−1.69	0.094

6MWD, six minute walking distance; LPHQ, Living with Pulmonary Hypertension Questionnaire; NYHA, New York Heart Association.

Only showing results where *p-value is <0.1.*

**Figure 3:**
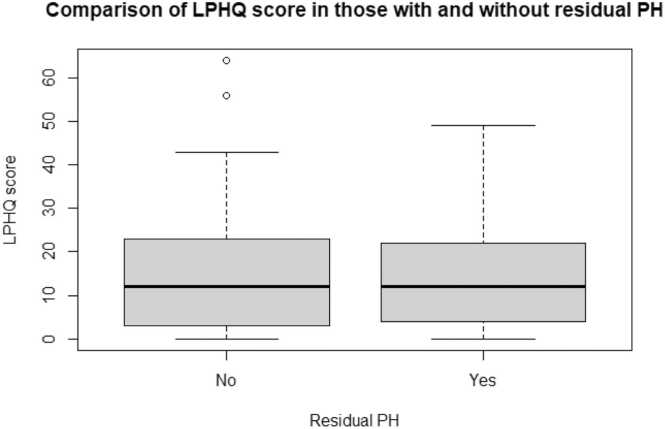
Comparison of quality of life in those with residual pulmonary hypertension 6 months after therapy. An LPHQ score of 0 represents high quality of life, LPHQ score of 60 represents very poor quality of life. LPHQ, living with pulmonary hypertension questionnaire.

The groups where residual PH was present at 6 months after treatment did not significantly differ in LPHQ scores at 6 months (Figure 3). Similarly, those with residual exercise intolerance did not differ in LPHQ scores from patients who made a complete hemodynamic recovery.

## Discussion

In this study, we found similar QoL both before and after treatment in 2 separate groups of CTEPH patients undergoing either PEA or BPA. Multiple functional parameters were associated with QoL at both time points. QoL after treatment improved regardless of the presence of residual PH.

In this cohort, baseline LPHQ scores were comparable to pre-therapy LPHQ scores seen in other studies.^19^, ^22^ We saw few statistically significant differences between baseline hemodynamic, or exercise tolerance parameters between patients scheduled for PEA or BPA. This is in line with what Ravnestad et al.^25^ observed, yet also in contrast to Tamada et al.^15^ who had observed a higher baseline mPAP and PVR in PEA patients in their cohort. This could be reflective of the increasing overlap between patients that are eligible to be treated with either PEA or BPA. Another explanation could be the different PROM used, as there has been established standard for QoL questionnaire in PH. Regardless of the baseline group differences, both studies observed comparable QoL improvements in both groups in line with our study.^15^, ^25^

Basic functionality and perceived dyspnea – as measured in the 6MWD test, the Borg score and the NYHA score – correlated most strongly with QoL at baseline in this study. This finding is in line with studies in PEA where QoL correlated to the 6MWD score but differs in that age and hemodynamic parameters did not correlate to QoL in our this cohort.^6^, ^15^

Interestingly, baseline DLCO was a moderate predictor of QoL at 6 months. Previous literature has shown DLCO to be a predictor of residual PH,^26^ and has been argued to indicate distal vasculopathy not amenable to surgery. This could indicate that CTEPH patients with impaired DLCO may gain less from invasive therapy.^6^ This is supported by previous studies where DLCOc was correlated to clinical outcomes in CTEPH patients such as six minute walking distance and NYHA score, but not hemodynamic outcomes.^27^, ^28^ Furthermore in a separate study DLCOc was associated to non-responsiveness after BPA in CTEPH patients.^29^

At the 6 month follow up, similarly to the baseline correlations, the NYHA and Borg score correlated with QoL. In this group of treated patients, some hemodynamic parameters and peakVO_2_/kg appeared to correlate to QoL. This could indicate that once basic functionality or pulmonary circulation is (partly) restored, hemodynamic factors and exercise capacity start to influence QoL.

The change in QoL over time was associated to a relative improvement in peakVO_2_/kg. In contrast to previous studies in PEA, we did not observe an association between the change in PVR or 6MWD over time to QoL (where both the SF-36 and the CAMPHOR were used) (Table 4).^6^, ^30^

In mixed PH populations, PROMs have been shown to correlate to exercise capacity, hemodynamics, functional class and survival. The relationship between hemodynamics and the LPHQ score have already been observed to be variable.^20^ In this study, this was most notably seen by how an improvement of hemodynamics was associated to an improvement in QoL, yet the presence of residual PH at 6 months was not correlated to decreased QoL at 6 months. Studies in PEA patients using the CAMPHOR do observe this difference in QoL in patients with residual PH.^6^, ^31^ This may indicate that the CAMPHOR and LPHQ differ in their sensitivity to hemodynamic changes, that hemodynamics is only partly influences perceived QoL after treatment or that 6 months is too soon after treatment to definitively establish residual PH. The CAMPHOR has been more often applied in studies on PEA patients and the LPHQ more in pharmacological CTEPH studies.^20^, ^22^, ^31^, ^32^ Whether the difference in association to hemodynamics is due to the PROM itself or due to the difference in CTEPH populations that they have been applied to is unclear. In patients treated pharmacologically and/or with BPA, a mild correlation between 6MWD and PVR to QoL using the SF-36 was seen,^12^ though more studies comparing LPHQ and CAMPHOR in more diverse CTEPH populations would be needed to clarify the role of hemodynamics in QoL.

This study’s strengths lie in that this was a well-defined cohort of PEA and BPA patients treated in the same centers and who were compared using the same CTEPH validated QoL assessment tool. This allowed for a broad exploration using multiple different clinical parameters. Markers for dyspnea during basic functionality consistently correlated to QoL and hemodynamic parameters, though associated at certain points, did not conclusively dictate QoL. A limitation of this study is that the patient groups were not randomized. Operation risk due to comorbidities and whether lesions were proximal or distal affected the choice of therapy. Therefore the 2 therapy groups cannot be directly compared in this study. Furthermore the use of a subjective measurement tool as outcome has an inherent risk of bias such as recall bias, social desirability bias and patient variability in interpretation of the questions.More is to be learned about which PROM is most informative for both PEA and BPA patients and should be selected for future generalizability of QoL studies.

This study is a step towards better understanding the recovery perceived by patients after invasive therapy for CTEPH and understanding which clinical parameters play a role in this perceived improvement. This is particularly relevant as BPA becomes more established as a viable alternative and addition to PEA in a broader selection of patients. These results may help inform future patients and clinicians when deciding on which treatment modality to undergo.

## Author contributions

HJB, AA, SA, NB, AK: Wrote the protocol and set up the study. PS, NB, AK, TR, ES, AA: Operational aspects of the 2A3C study. PS, LV, AA, CT, HJB: Analysis and interpretation of data. PS, HJB: Wrote the first draft of the manuscript. EN, JA, HJB, AVN, LM, AW, MB, RL, JW, FO, TM: All contributed to collection of data, provided input into the interpretation of the data. All authors reviewed final version of the manuscript.

## Disclosure statement

The authors have no financial conflicts of interests to disclose.
