# Supplementary material for: Similar quality of life after balloon pulmonary angioplasty or pulmonary endarterectomy for CTEPH
Source: JHLT Open. 2025 Jan 31;8:100223. doi: 10.1016/j.jhlto.2025.100223 (PMC11935437; doi:10.1016/j.jhlto.2025.100223)
Supplement: Supplementary file 1 — Supplementary material [file mmc1.docx]

# Results of analysis where BPA patienst with 2 or less sessions have been excluded

**Revised Figure 1:**

**
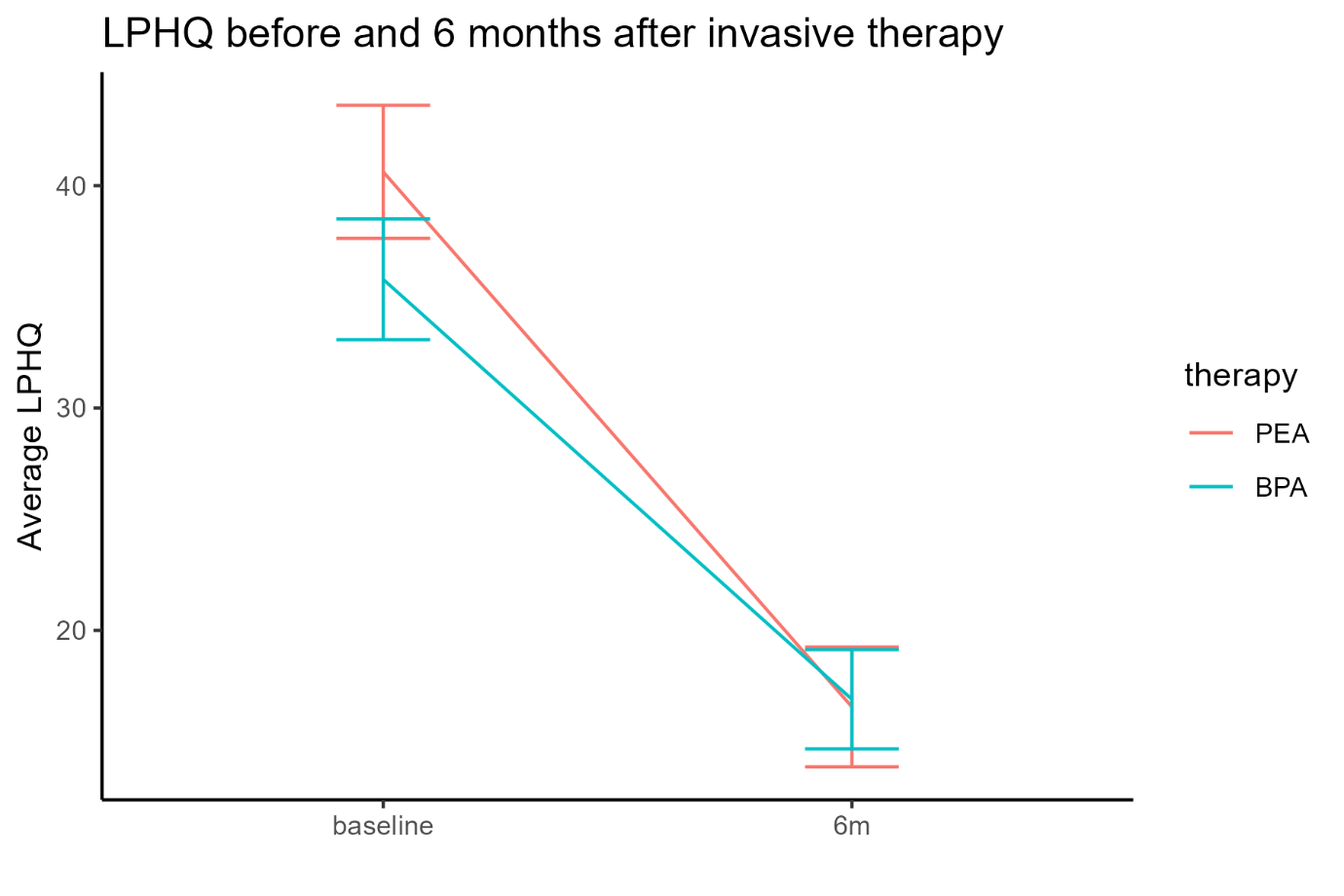
**

**Revised results from Table 2:**

**T-test showing difference LPHQ between BPA and PEA groups at baseline**

data: df2a3c$LPHQ by df2a3c$therapy

t = 1.1966, df = 84.674, **p-value = 0.2348**

alternative hypothesis: true difference in means between group PEA and group BPA is not equal to 0

95 percent confidence interval:

-3.2 12.8

sample estimates:

**mean in group PEA mean in group BPA**

**40.6 35.7**

**T-test T-test showing difference LPHQ between BPA and PEA groups at 6 months**

data: df2a3c$LPHQ_6m by df2a3c$therapy

t = 0.52925, df = 66.615, **p-value = 0.5984**

alternative hypothesis: true difference in means between group PEA and group BPA is not equal to 0

95 percent confidence interval:

-5.1 8.9

sample estimates:

**mean in group PEA mean in group BPA**

**15.7 13.9**

**Revised Figure 3 with corresponding result of analysis:**

**
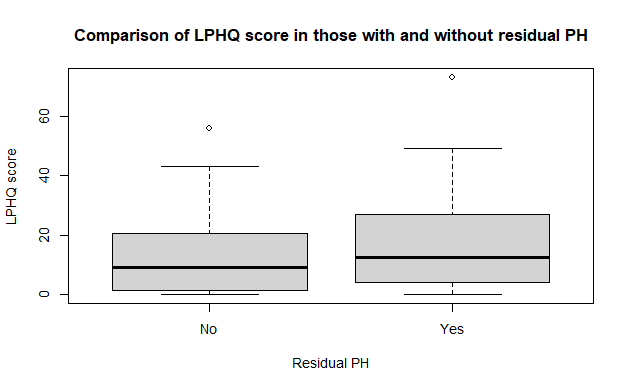
**

data: LPHQ_6m by rest_PH_6m

t = -1.0418, df = 64.938, **p-value = 0.3014**

alternative hypothesis: true difference in means between group No and group Yes is not equal to 0

95 percent confidence interval:

-11.075234 3.481957

sample estimates:

**mean in group No mean in group Yes**

**13.08571 16.88235**
