# Supplementary material for: Similar quality of life after balloon pulmonary angioplasty or pulmonary endarterectomy for CTEPH
Source: JHLT Open. 2025 Jan 31;8:100223. doi: 10.1016/j.jhlto.2025.100223 (PMC11935437; doi:10.1016/j.jhlto.2025.100223)
Supplement: Supplementary file 2 — Supplementary material [file mmc2.docx]

**Supplementary Figures and Tables**

Table 1: Complete baseline table with all clinical parameters.

|  | **BPA** | **N** | **PEA** | **N** | **t-value** | **p-value** |
| --- | --- | --- | --- | --- | --- | --- |
| *Demographics* |  |  |  |  |  |  |
| **Age at time of consent** | **67.1 (9.9)** | **54** | **62.7 (11.1)** | **45** | **2.08** | **0.04** |
| Gender |  |  |  |  |  |  |
| BMI (kg/m^2^) | 26.1 (4.5) | 54 | 27.5 (4.5) | 45 | -1.5 | 0.14 |
|  |  |  |  |  |  |  |
| *Comorbidities* |  |  |  |  |  |  |
| COPD | 4% | 48 | 5% | 41 |  |  |
| Hypertension | 22% | 48 | 33% | 41 | -1.27 | 0.207 |
| Ischemic heart disease | 9% | 48 | 4% | 41 | 0.93 | 0.355 |
| Kidney disease | 4% | 48 | 7% | 41 | -0.67 | 0.507 |
| Other | 45% | 48 | 4% | 41 | 0.54 | 0.588 |
| None | 38% | 48 | 31% | 41 | 0.74 | 0.464 |
|  |  |  |  |  |  |  |
| *Medication* |  |  |  |  |  |  |
| **Riociguat** | **2%** | **48** | **13%** | **41** | **-2.12** | **0.039** |
| Sildenafil | 4% | 48 | 4% | 41 | -0.2 | 0.841 |
| VKAs | 31% | 48 | 47% | 41 | -1.61 | 0.111 |
| **NOAC** | **69%** | **48** | **47%** | **41** | **2.29** | **0.024** |
| Loop diuretics | 18% | 48 | 16% | 41 | 0.35 | 0.73 |
| Aldosteron receptor antagonists | 5% | 48 | 2% | 41 | 0.85 | 0.398 |
| Others | 5% | 48 | 11% | 41 | -1 | 0.32 |
|  |  |  |  |  |  |  |
| *Clinical, lab, LFT and 6MWD* |  |  |  |  |  |  |
| LPHQ (score out of 105) | 36.6 (18.9) | 54 | 40.6 (20.1) | 45 | -1.03 | 0.31 |
| Δ LPHQ 6 months after therapy | 21.3 (15) | 27 | 25.7 (18.1) | 35 | -1.04 | 0.3 |
| NYHA score | 2.4 (0.6) | 52 | 2.6 (0.6) | 45 | -1.45 | 0.15 |
| DLCOc (mmol/(min*kPa)) | 6.3 (2.1) | 30 | 6.4 (1.9) | 35 | -0.11 | 0.91 |
| DLCOc (%pred) | 70.1 (16.4) | 46 | 72.5 (14) | 39 | -0.74 | 0.46 |
| CRP (ml/min) | 5.4 (7.6) | 51 | 6.2 (10.3) | 38 | -0.38 | 0.7 |
| eGFR (ml/min) | 73 (13.5) | 54 | 71.3 (13.8) | 45 | 0.63 | 0.53 |
| NTproBNP (ng/L) | 1063.4 (1517.9) | 54 | 1170.7 (1610.6) | 43 | -0.33 | 0.74 |
| 6MWD (m) | 438.2 (118.3) | 47 | 444.2 (93.9) | 30 | -0.25 | 0.81 |
| 6MWD (%pred) | 84.9 (21.6) | 17 | 82.3 (13.7) | 20 | 0.42 | 0.68 |
| Borg score at end of exercise | 5.5 (2.3) | 46 | 5.4 (2.5) | 28 | 0.17 | 0.86 |
|  |  |  |  |  |  |  |
| *Right heart catheterisation* |  |  |  |  |  |  |
| *sPAP* (mmHg) | 67.1 (23.8) | 54 | 71.4 (22.8) | 45 | -0.92 | 0.36 |
| *dPAP* (mmHg) | 23.9 (9.7) | 54 | 25.6 (8.8) | 45 | -0.95 | 0.34 |
| mPAP (mmHg) | 39.3 (14.4) | 54 | 41.7 (13.2) | 45 | -0.84 | 0.4 |
| sRAP (mmHg) | 10 (4.4) | 44 | 11.3 (4.4) | 40 | -1.39 | 0.17 |
| dRAP (mmHg) | 5.7 (5.5) | 44 | 5.2 (4.6) | 40 | 0.41 | 0.68 |
| mRAP (mmHg) | 6.7 (4.1) | 54 | 7.4 (3.8) | 45 | -0.87 | 0.39 |
| sRVP | 66.5 (24) | 52 | 69.8 (21.8) | 42 | -0.71 | 0.48 |
| dRVP | 6.7 (5.3) | 52 | 6.7 (5.3) | 42 | -0.06 | 0.95 |
| Cardiac output | 5.3 (1.6) | 54 | 5.2 (1.6) | 45 | 0.38 | 0.7 |
| Cardiac Index (l/min) | 2.6 (0.7) | 54 | 2.6 (0.7) | 45 | 0.67 | 0.51 |
| PAWP (mmHg) | 10.5 (3.5) | 54 | 11 (2.7) | 45 | -0.77 | 0.45 |
| PVR (dyn/s/cm5) | 497.2 (314.4) | 54 | 585.9 (411.7) | 45 | -1.19 | 0.24 |
| SvO2 (%) | 65.9 (10.2) | 52 | 65.4 (7.9) | 45 | 0.29 | 0.77 |
|  |  |  |  |  |  |  |
| *Cardiopulmonary excercise testing* |  |  |  |  |  |  |
| **peakVO2 (%pred)** | **71.4 (23.5)** | **47** | **61.5 (21.2)** | **42** | **2.09** | **0.04** |
| peakVO2 (ml/min ) | 1349.5 (558.9) | 48 | 1280.1 (513.7) | 41 | 0.61 | 0.54 |
| peakVO2 (ml/kg/min) | 16.4 (5.9) | 47 | 14.7 (5.7) | 40 | 1.34 | 0.18 |
| Load max (watts) | 105.1 (46.6) | 47 | 99.1 (52.7) | 42 | 0.56 | 0.58 |
| **Load_max (%pred)** | **87.1 (39.9)** | **43** | **63 (32.1)** | **40** | **3.03** | **0.001** |
| Heart rate max (bpm) | 130.4 (24.4) | 47 | 133.5 (21.3) | 41 | -0.64 | 0.53 |
| Heart rate max (%pred) | 85.2 (14.8) | 47 | 84.6 (12.5) | 41 | 0.2 | 0.84 |
| **RERmax** | 3.4 (16) | 48 | 1 (0.1) | 41 | 1.02 | 0.31 |
| O2pulse max (ml) | 10.4 (3.4) | 47 | 9.5 (3.4) | 41 | 1.29 | 0.2 |
| **O2pulse max (%pred)** | **83.8 (29.5)** | **47** | **70.5 (25.4)** | **41** | **2.27** | **0.03** |
| EqCO2_AT | 44 (11.8) | 46 | 47.3 (10.6) | 37 | -1.33 | 0.19 |

Supplementary table 2: Comparison of the change of clinical variables from baseline to 6m follow-up between therapy groups.

|  | **BPA** | **N** | **PEA** | **N** | **t-value** | **p-value** |
| --- | --- | --- | --- | --- | --- | --- |
| **Δ DLCOc** | **-0.1 (0.6)** | **13** | **0.3 (1.1)** | **25** | **-1.72** | **0.09** |
| Δ PERpredDLCOc | -2.5 (5.4) | 13 | -0.2 (7.2) | 26 | -1.11 | 0.27 |
| Δ NTproBNP | 256.6 (1151.9) | 29 | 666.3 (1440.3) | 34 | -1.25 | 0.21 |
| Δ sixMWD | -46.6 (44.9) | 20 | -63.1 (47.6) | 12 | 0.97 | 0.34 |
| Δ 6MWD (%pred) | -12.8 (4.3) | 5 | -11.9 (5.2) | 7 | -0.34 | 0.74 |
| Δ Borg score | 1.1 (3) | 19 | 2.4 (2.1) | 10 | -1.42 | 0.17 |
| **Δ mPAP** | **10.3 (8.7)** | **30** | **16.9 (11.4)** | **36** | **-2.65** | **0.01** |
| **Δ PVR** | **235.2 (253.2)** | **30** | **375.7 (373.5)** | **35** | **-1.8** | **0.08** |
| **Δ sRVP** | **16.3 (14)** | **30** | **28.4 (21)** | **33** | **-2.73** | **0.01** |
| Δ VO2max | 233.8 (265.2) | 19 | 308.4 (392.8) | 31 | -0.8 | 0.43 |
| Δ peakVO2 (%pred) | -13.5 (13.9) | 19 | -15.3 (14.4) | 32 | 0.45 | 0.65 |
| **Δ EqCO2_AT** | **5.6 (6.1)** | **18** | **11.8 (8.7)** | **28** | **-2.84** | **0.01** |
| Δ EqCO2_max | 7.8 (8.1) | 14 | 12.3 (16.6) | 30 | -1.21 | 0.23 |

Supplementary table 3: Clinical variables at 6 months correlated to LPHQ at 6 months

| **Correlation 6 month variables to LPHQ at 6 months** | **Correlation** | **p-value** | **N** |
| --- | --- | --- | --- |
| NYHA | 0.439 | 0.001 | 82 |
| Borg score after 6MWD | 0.486 | 0.001 | 65 |
| Right atrial pressure (diastolic) | 0.472 | 0.001 | 66 |
| Pulmonary vascular resistance | 0.425 | 0.001 | 82 |
| Six minute walking distance | -0.403 | 0.001 | 65 |
| peakVO2 (ml/kg/min) | -0.351 | 0.002 | 73 |
| Cardiac index | -0.317 | 0.004 | 80 |
|  |  |  |  |
| BMI | 0.253 | 0.042 | 65 |
| DLCOc (%pred) | -0.309 | 0.049 | 41 |
| C-reactive protein | 0.377 | 0.002 | 65 |
|  |  |  |  |
